# Another Century

**Published:** 1900-08-15

**Authors:** A. W. Diack


### ANOTHER CENTURY.

BY A. W. DIACK, D.D S.

Some one has said that, in order to be a consistent optimist, one should look continually upon the future and should view the present only in the light of dreams of future perfection. This is almost axiomatic in its force and simplicity, for, as we gaze at our present conditions in almost any of our relations with our fellow-man, we are jarred and shocked at life's incongruities and disheartened by the immediate contact with base ideals. We are apt to place too much importance on specific instances of evil, and, when we let these events influence our opinions, we are forced to conclusions which are not in accord with progress. A pessimism settles upon us, discouraging and disheartening. But, when we take a broader view, looking back upon the past, seeing the things which have been done, and forward into the future with an appreciation of the possibilities which the past experience has given us, we can be naught else but optimistic.

So, when we consider our profession to-day, it will be well if we gaze at the past, and, discarding the evils of the present, dream of the day that is to come.

I shall take no time to rehearse the old, old story of how our work was first performed by the blacksmiths and the barbers, nor how the acme of professional ambition was reached when small cavities of decay were filled, when the tooth with the exposed nerve was condemned to destruction and when the carving of plates from ivory was the perfection of mechanical dentistry. This is perfectly known to us all.

But in passing this by as trite, it is by no means proper to pass the men of that period with small mention if we would learn properly the idea of professional life. Bitter were the prejudices and long were the struggles in the early days before the advent of the noble men into dentistry who appreciated the fact that dentistry depended primarily upon knowledge and skill, instead of skill only. Appreciating this they could not but take heed of the logical conclusion of this appreciation, i. e., that the knowledge of all was greater than the knowledge of any one. These men were also in possession of the idea that their lives in this world endowed them with a duty to humanity. They had learned that service was the only badge of freedom, and that they realized their better selves most when they were of most service to their fellow-men.

Prejudice, bitter though it was, and struggles, prolonged as they may have been, could not long withstand the forces of knowledge and the duty of social service, and as a result, we see men meeting on this common ground and realizing a common end. What else could be produced by men in the same walk in life, imbued with the same ambitions, than societies for their future benefit?

It is this view of the past which we should ever keep in mind, when we consider the passing of the times that were and are, and look into the future with the hope that by our actions in the present we may be moving toward the truth. In scientific progress the coming century certainly appears at its birth to be fraught with great possibilities. When we cast a backward glance over the last half century and realize what has been done in the scientific development of dentistry; when one sees the substitution of rational ideas for the baldest empiricism; when it is seen that we are nearly on the threshold of practical application of newly discovered natural laws, it is no wonder that the future seems so bright with promises of untold advancement.

More and more the intimate relations of dentistry to medical science is asserting itself, and more and more eager are the members of the profession to ally themselves to the mother science. Dental thought, instead of being solely occupied with the discovery of the best means of overcoming the ravages of caries, is directing its energies to the prevention of the process. The discovery of the cause has forced the search after means of removing the cause. Not only has the future century the problem of the prevention of the immediate cause of disease, but it also has the discovery of predisposing causes, and at no distant day will the relations of the housing, sanitation and preparation of foods be considered as important in the prevention of dental disorders as tooth brushes and antiseptics are considered now. Sad as it may be, it seems nevertheless to be true that, as civilization advances, the new physical environment of man produces changes which have a decidedly deleterious effect upon the body. Mental activities, which leave no inclination to physical development; unscientific housing; neglect of surroundings regarding sanitation and the perpetuation of the race by parents physically weak, are surely producing conditions which conduce to dental disorders. This is a problem for the future and one which, as the next century grows older, will assert itself vigorously. Scientific prophylaxis, having to do with heredity and environment, will be the broadest field of medical investigation.

Standing on the foreground of past accomplishment, and knowing how much our progress has been due on the part of our predecessors to the noble striving after something better, we can see standing out from all the influences which made a profession of dentistry, the dental society and the dental college. From the first has come the power which has created dental laws, and from the dental college have come the men educated in professional ideals who have been the bone and sinew of the dental societies.

The advancement in dentistry has been too potent to allow us to tolerate for one moment the idea that it has deteriorated and its practitioners have again assumed the position of prejudice and struggle. But, however great our advancement and seeming progress, the question arises now as the manifold problems are presented to us, have there not occured changes in professional activity which leads one to wonder whether we are progressing as rapidly as we should, and whether we are not still clinging to those things which, having served their purpose in the past, have now been out grown. In everything there is either growth or decay. Growth, so long as there is an adaptation to, and a conquering of the environment; decay, when the adaptability has ceased and environment crushes the vitality that is no longer able to assert itself. Men and institutions alike are amenable to this law. That which is good persists perhaps in other forms, while that which is bad passes away.

Associations of men with a common aim crystallize their beliefs and hopes in some formulated manner. This formula is best adapted to the conditions in which the association is to labor. But the times come when the conditions change, and then the formula must be changed or relegated to make way for something more in accord with changed conditions. Too often it is that formulæ are retained so long as to bring about a destruction of the association. Creeds and constitutions, true though they may have been, will some day be lifeless forms. As men change, the institutions and creeds must change. Institutions were made for man, not man for institutions. And now, in the last days of the nineteenth century, we find conditions markedly different from those in which the first dental societies had their beginning.

It should be the aim of our dental societies to adapt themselves to these new conditions, for only by such action will the permanency of the association for usefulness be established. By a more liberal interpretation of our Code of Ethics no doubt a great deal could be accomplished, but a much more lasting benefit would be conferred upon the profession by a new and broader formula of aims and standards. By such action on the part of dental societies the future progress of the science is assured. By lack of such adaptation to the present conditions, advancement will be retarded and bitterness will spring up which in the future will be an obstacle to the attainment of professional ideals.

We no longer, as a general rule, have as competitors in professional life uneducated men. The college course is beginning to make itself felt in the personality of the profession, and the rules of action which were required in the past have only the effect of arousing opposition.

Next to the dental society, and perhaps of more importance as a formative professional influence in the next century, is the dental college. Herein lies the hope of the future. To the source of any stream one must look for the purest water. Although much may be said in criticism of the modern dental school and their methods, boasts and real objects, it must be admitted that they are improving in character. The great danger is in the commercial spirit which rules them. In education we can afford to be altruistic in our conduct. Not only can we afford to, but we are obliged to be if we would gain all the blessings of education which come as culture, refinement and appreciation of public duty. When commercialism intrudes itself it is felt as a blight by all who come under its influence. But even the colleges of the lower grade are forced by the dignity, scholarship and high ideals of the best schools, to follow after in the same path, and, slow though the advancement seems, it is at least advancement.

The future of the profession depends upon the personality of the man, the ideals he worships and the education which he receives. The standing of the professional man in the future will not be based upon the same conditions which have served in the past. The time is passing rapidly when the mere title of "Doctor" or the mere possession of a degree from an institute of learning is considered as giving entree to the band of noble men who are really serving humanity. In the future it will be the man who does the work by truly serving the community in his field.

The next century does indeed look bright, not only for the profession itself so far as scientific progress is concerned, but for the lives of those in the profession, who, possessing ideals, are ambitious and energetic. Many are the obstacles to be overcome and disappointing are the efforts at betterment, yet with honest effort to serve with knowledge and skill our fellow-men, the true happiness of living may be attained in our life work. Thus may we say with Holmes:

Build thou more stately mansions, oh, my soul!
  As the swift seasons roll.
  Leave thy low-vaulted past;
Let each new temple, nobler than the last,
Shut thee from heaven with a dome more vast;
  Till thou at length art free,
  Leaving thine outgrown shell by life's unresting sea.

---

Owing to the absence of Dr. Walton, Dr. Blackmarr was asked to open the discussion on the papers.

Dr. Blackmarr: Mr. President, I like to see a discussion opened without notes but I expected my wife to be here and I do not have that freedom of speech when I know she is anywhere near.

One year ago as I finished my labors and pleasant duties of president of this Association, Dr. Fowler said to me, "now Blackmarr, we don't want to hear a word from you in five years. Ex-presidents are expected to lie down and keep quiet and not even to be heard of again."

So, that when our president wrote me asking me to discuss Dr. Fowler's paper I was delighted to have the chance to get back at Dr. Fowler. I expected, of course, to get even with him but as I know how sure he is to go to Indianapolis next year to the Tri-State meeting and also as I read his paper over and noticed his criticism on the dental colleges I felt rather sorry for him on the whole, especially after the college teachers and dental examiners get through with him. This part of Dr. Fowler's paper I will leave for those men to open discussion on, and I truly hope it will be thoroughly discussed. With your permission I will take a few of Dr. Fowler's sentences and consider them a little further. "Organization." I am delighted to know that Dr. Fowler used this word and believes in organization. I am glad to believe that the "future dentist" will belong to an organization of dentists. Co-operation is so far ahead of competition in all matters concerning the profession's labor and brotherhood in general, and it does seem to me that we each should encourage others to join our State Association and make this organization more subsurvient to the good of all members in more ways than now. Another sentence in Dr. Fowler's paper says, now a dentist or preacher, there is no calling so noble as one which teaches man how to live better than he is living. There is not a calling higher than teaching man how to have health of soul. I expect the reason why the ordinary orthodox preacher is held in such repute is because he does not teach actual facts. The more enlightened preachers to-day recognize the truth of evolution and teach that heaven is not a *place* to jump into and be happy, but rather a *condition* into which man can evolve himself. That right-doing always comes out right and that wrong-doing brings ruin and wrong results. And now I think preachers and the future dentist should be more alike in regard to teaching the people how to live so as to avoid the results of sin, and by the future dentist how to live and take care of the dental apparatus so as to avoid the results of decayed and malposed teeth. Orthodox belief in religion and dentistry both have the idea of some one *else* than ourselves or our patients do the work of saving us from utter ruin, and in dentistry the dentist is expected to save the teeth and has taught his patients so, while the fact is, that we have to learn how to save ourselves by knowledge gained by observation, experience, advice, etc. And so I believe the future dentist should teach his patients how to keep their teeth and avoid trouble. I believe the most successful lawyer is one who advises his clients to avoid trouble. I believe the most successful physician is one who tells his patients the cause of their illness and how to avoid it in the future. I believe the most successful dentist in the future will be one who will teach parents to bring their children to the dentist early and often, and he will see that the child breathes normally (through the nose), and so avoid the child becoming a mouth-breather with its many bad results. He will teach the child to properly care for its teeth. He will arrest any decay in its incipiency, retaining the temporary teeth until they are normally lost. The second set are guided (if necessary) into their normal position and occlusion—so wonderfully shown by Prof. Angle—which is of far more importance in preventing decay, etc., than is usually considered. This child now has a superb chance to be immune from decay, etc. Now I believe the lawyer, preacher, physician or dentist who avoids trouble for his intelligent patrons will be given higher fees than the one who does otherwise.

Dr. Hoff: Several things have been opened up by these papers and the discussion by Dr. Blackmarr. One statement of Dr. Fowler I will read, it is the sentence which reads, "You may argue that some of these same students make better and more practical dentists than many with the highest literary attainments," etc. The man best qualified to make a practical dentist. It requires a very versatile man to make the future dentist, he should not be simply a mechanic; he must be more than that. He must be more diverse than to be able to make a rubber plate, amalgam filling, etc. He must be able to diagnose diseased conditions which manifest themselves in the mouth and which are often systemic in character, and are oftentimes very puzzling and not successfully treated by any local treatment, however skilfully applied.

For the good of the public health the dentist of to-day comes far short of accomplishing what he should; we are not saving people's teeth in any adequate measure. This is a matter which I have thought a great deal about in viewing the future, when asked to advise young men as to the best methods for building up a practice, how to best accomplish it. I tell students to select a place where they think they want to live and then go there and equip themselves as well as their means will permit and use their utmost endeavors to serve the people as well as they possibly can.

It is not only in the direction of advancement in literary qualifications for which we are to look for the future dentist. He may be a scholar, very learned, and have a very high standing as a literary man, and yet be a very poorly qualified dentist, entirely unfit to take up the general practice of dentistry and make a success of it, so far as doing justice to his patients is concerned.

Many such examples are found in our colleges; some of our best students make most disastrous failures as practitioners. It is very difficult to harmonize these conditions and define universal standards as to necessary qualifications for an adequate dental education. I do not know of any better way than to make the standard as nearly ideal as possible, and then allow each to take his chances as to whether he is going to succeed in his vocation or not.

In regard to the criticism that the entrance requirements are not strictly observed, I am in a position to know as much about that as Dr. Fowler as I have had experience personally; it is one of my duties in my connection with the University to pass upon qualifications of men entering that institution. I am also familiar with the practice in almost all of our schools and also as to the attitude which different schools take upon this subject.

I know that there is reason for criticism, I do not say this in the way of defence of colleges, or as contradicting the statement of Dr. Fowler. The large majority of teachers in the schools are making an honest endeavor to look into the qualifications of students more carefully than ever before in our history. Not only the literary but the technical ability, natural or acquired, is investigated as never before. While some of you may not believe this, it is true, you will see the results in properly qualified men, good men who will hold their practices. I am very confident that while it is true that dental schools are to-day perhaps as never before striving, as the essayist says, to build up their schools in numbers and increase as much as possible the revenue, there is also going along with that an elevation of the standard of entrance and passing requirements in all the shools throughout the country. Even the schools which have been the subjects of the most severe criticism are adopting this method; they realize the necessity of taking this stand; they are compelled to take it. Poorly qualified students are more trouble and expense to teach, and this compels them to raise their standards. One-half dozen students who are poorly qualified will consume more time of the teacher than all the balance of the class. Such students require the instructor to sit down beside them and drum things into them, and where there is a class of two or three hundred it is impossible to do this; the work must be done in a wholesale way, class exercise, lectures, etc. And since these students are really a burden to the school, these things as a matter of course will eventually work themselves out.

Dr. Loeffler: There is one point which is really brought out in both papers which I would like to touch upon for a few moments. Literary requirements. Things have been brought up that would in a measure reflect on the work being done in our colleges and universities. As practical men or as regards work in a practical way we have a standing here in this country that is perfectly surprising, and it is brought to our notice more pointedly by those who have traveled abroad and have had occasion to find out this fact and noticed with what repute our American dentists are held by those in Europe. And this is brought about in no other way than by our schools which are so much better than those of the foreigners. Now, if this is surprising to intelligent people, it certainly must be more so to those who have not given it any special thought, and goes to prove that as practical men we have a higher standing here than in any other country.

If any improvement is needed it is in a literary way. In all colleges in Europe the entrance requirements are high. If a student wishes to take up the profession of law, medicine or any other, it is not a question of passing an examination but he must hold a diploma or at least must be a graduate from what may be called an academy, which is nearly equal to the university diploma. They must be fully prepared in a literary way in order to take up any profession.

Some criticism has been made in regard to the entrance requirements—perhaps it is true. I have some experience in regard to that matter myself. I also positively know that within the past ten years the progress that has been made in this direction is wonderful. The faculty, as a rule, are broader in their ideas as to necessary preliminary requirements. I remember very distinctly after finishing my literary course in the university and then taking up the dental course of feeling as if I had wasted my time in taking the literary work, but I am not sorry now that I took the work. I think, as the essayist has stated, the future dentist will be required to hold a diploma from a university or academy to enter the dental colleges. In spite of the criticism, I positively know that the faculties in some schools at least are making every effort to elevate that standard.

Dr. Root: The future dentist does not depend entirely upon college education. If he is able to pass the State laws in some States he is allowed to practice, and you all know how easy these examinations are to pass; he must know a little something about anatomy and several other subjects which he can study up. Why not change the law requiring men to go to a college? If the State examinations are below the standard of the colleges students will quit going to school and pass the examinations and get their permit to practice; I know of two or three examples where they have done so and not gone to college. So I say if we want to raise the standard, make the requirements of the Board of Examiners equal to the college examinations.

Dr. Woolsey: I think it is an elegant point made by Dr. Root. In the State of Minnesota no one is allowed to take the examination unless he presents a diploma from an authorized college, and then he is obliged to pass the State examination which cuts out the poorer students and brings only the better dentists into the State. The future dentist in order to be elevated must be a graduate of a reputable dental college.

*(To be Continued.)*

